# Single‐cell transcriptomics provides new insights into the role of fibroblasts during peritoneal fibrosis

**DOI:** 10.1002/ctm2.321

**Published:** 2021-02-26

**Authors:** Jinhua Zhang, Yuxian Chen, Tufeng Chen, Bin Miao, Zuofu Tang, Xiao Hu, You Luo, Tong Zheng, Ning Na

**Affiliations:** ^1^ Department of Kidney Transplantation The Third Affiliated Hospital of Sun Yat‐sen University Guangzhou China; ^2^ Department of Joint Surgery The Third Affiliated Hospital of Sun Yat‐sen University Guangzhou China; ^3^ Department of Gastrointestinal Surgery The Third Affiliated Hospital of Sun Yat‐sen University Guangzhou China

**Keywords:** fibroblasts, peritoneal fibrosis, receptor‐ligand interactions, single‐cell transcriptomics

## Abstract

**Background:**

The contributions of various types of cell populations in dialysis‐related peritoneal fibrosis are poorly understood. Single‐cell RNA sequencing brings single‐cell level resolution to the analysis of cellular transcriptomics, which provides a new way to further characterize the distinct roles and functional states of each cell population during peritoneal fibrosis.

**Methods:**

Single‐cell transcriptomics from normal peritoneal tissues of six patients, from effluent of patients with short‐term peritoneal dialysis (less than 2 weeks, *n* = 6), and from long‐term peritoneal dialysis patients (more than 6 years, *n* = 4) were analyzed.

**Results:**

We identified a distinct cell component between samples among different groups. Functional analysis of the differentially expressed genes identified cell type specific biological processes relevant to different fibrosis stages. Well‐known key molecular mechanisms participating in the pathophysiology of peritoneal fibrosis were vitrified, and some of them were found to be restricted to specific cell types. Gradually growing enrichment of PI3K/AKT/mTOR pathway and impairment of oxidative phosphorylation in mesothelial cells and fibroblasts were found from healthy control, short‐term dialysis, to long‐term dialysis, respectively. The fibroblasts’ population obtained from the patients, who received peritoneal dialysis, showed a functional characteristic of immune‐chemotaxis and immune response, which was characterized by broadly significant increase in the expression of interleukins, chemokines, cytokines, and human leukocyte antigens. Furthermore, we described the intercellular crosstalk networks based on receptor‐ligand interactions, and highlighted a central role of fibroblasts in regulating the key mechanisms of peritoneal fibrosis through crosstalk with other cells.

**Conclusions:**

In summary, despite describing information for fibrogenic molecular mechanisms in the resolution level of individual cell populations, this work identifies the significant functional evolution of fibroblasts during peritoneal fibrosis. This study also reveals the intercellular receptor‐ligand interactions in which the fibroblasts serve as a major node, eventually providing new insights into the role of fibroblasts during disease pathogenesis.

## Graphical abstract

1. Landscape dynamics of cell populations during dialysis‐related peritoneal fibrosis.
2. Description of pathophysiology in peritoneal fibrosis at single‐cell resolution.
3. Elucidation of receptor‐ligand interaction networks between fibroblasts and immune cells.

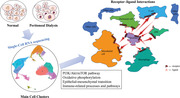

**Abbreviations**
CPM, coefficient of variation of the counts per million; DAVID: Database for Annotation, Visualization and Integrated Discovery
DC: dendritic cell
DEGs: differential expressed genes
EMT: epithelial‐mesenchymal transition
ESRD: end stage renal diseases
GO analysis: Gene Ontology enrichment analysis
GSEA: Gene Set Enrichment Analysis
HLAs: human leukocyte antigens
PD: peritoneal dialysis
PF: peritoneal fibrosis
scRNA‐seq: single‐cell RNA sequencing

## 1 INTRODUCTION

Peritoneal dialysis (PD) has been one of renal replacement treatments and widely used in patients with end‐stage renal diseases (ESRD).^1^ Although PD has several advantages compared with hemodialysis, long‐term PD triggers peritoneal fibrosis (PF) which progressively leads to histological change to peritoneal membrane and reduces membrane function, invariably resulting in ultrafiltration failure.^2^ By now, PF is still the main complication of PD and the leading cause for its discontinuation. However, the detailed molecular mechanisms underlying the development and progression of PF have not been entirely understood.

The normal peritoneal membrane is lined by a monolayer of mesothelial cells, which are supported by a sub‐mesothelial compact area containing fibroblasts, immune cells, lymphatic vessels, and a dense network of capillaries required for solute exchange.^3^ Long‐term PD results in the histological and functional changes of the peritoneal membrane, which was characterized by accumulation of fibroblasts.^4^, ^5^ Although it has long been described that fibroblasts provide only structural framework for tissues, it is newly identified that fibroblasts actually locate at the center of tissue homeostasis and serve specialized functions in fibrotic diseases.^6^ Noticeable diversity in the phenotypes of fibroblasts is observed even in the same disease.^7^ Meanwhile, numerous studies demonstrated that many elements of the innate and adaptive immune responses participate in the differentiation of fibroblasts.^8^, ^9^, ^10^, ^11^ The cellular composition is complex in the peritoneum environment; additionally, there exist diverse phenotypes and functions of fibroblasts. Therefore, it warrants further research on the function of fibroblasts and their interaction with the other cells during the progression of PF.

As each cell population in tissues has a distinct transcriptome, previous “transcriptome” using bulk tissues was limited by changes in single cell resolution. Single‐cell RNA sequencing (scRNA‐seq) is a novel technique to estimate the expression levels of thousands of genes in individual cells,^12^ which offers an unprecedented opportunity to resolving cell‐type contributions in tissues, with transcriptome information at a single‐cell level.^13^, ^14^ In our current study, we analyzed scRNA‐seq data of peritoneal cells obtained from normal peritoneum and the PD patients. We focused on fibroblasts in order to further elucidate the role of fibroblasts involved in this disease at a single‐cell resolution level.

## 2 METHODS AND MATERIALS

### 2.1 Collection of clinical samples and scRNA‐seq

Briefly, the scRNA‐seq dataset involved normal parietal peritoneal tissues obtained from six patients who underwent laparoscopic inguinal hernia repair, PD fluid samples from six ESRD patients received PD less than 2 weeks, and PD fluid samples from six ESRD patients received PD more than 6 years. Our study was approved by the Research Ethics Committee (IRB Number: [2020]02‐166) of the Third Affiliated Hospital of Sun Yat‐sen University, China. Contents were obtained from all the patients. All the patients received treatment at the investigators center, the Third Affiliated Hospital of Sun Yat‐sen University, China. The detailed procurements of collecting clinical samples, tissue dissociation and single‐cell isolation, single‐cell capture, cDNA library preparation, barcoding and sequencing, were described in the main text and supplementary materials of a previous publication by Si et al.^15^ In brief, the normal parietal peritoneal tissues were cut into pieces (about 4 mm^2^) and digested with digestion solution. Cold HBSS was used to stop samples reaction. A 70 μm cell strainer (Falcon) was applied to filter suspension for pellets, followed by incubation with red blood cell lysate and resuspension in phosphate‐buffered saline containing 0.4% BSA. Then, a single‐cell suspension was generated. Cell pellet from the effluent collected within 1 h of the PD patient's dialysate outflow was obtained after centrifugation and incubated with RBC lysis and resuspended in PBS containing 0.5% BSA for single‐cell suspension. The scRNA‐seq was performed with 10× Genomics using the Chromium Single Cell 3′ Gel Bead and Chip and Library Kits. Quality control, sample demultiplexing, barcode processing, and single cell 3′ gene counting were performed by using Cell Ranger Single Cell Software Suite (v3.0).

### 2.2 Cell typing by Uniform Manifold Approximation and Projection algorithm

First, the scRNA‐seq dataset used was obtained from a previous study,^15^ which has been uploaded to Gene Expression Omnibus (GSE130888). All the data from normal peritoneum and PF patients were analyzed collectively. The “Seurat package” of R software was used to perform preliminary analyses of scRNA‐seq data and segregate all cells into distinct clusters. To identify the cell type for each cluster, we detected gene markers for 15 distinct cell clusters using the “FindMarkers” function in Seurat package (v3.0.1), then we submitted the gene markers (top300) to Enrichr (https://amp.pharm.mssm.edu/Enrichr) for annotating cell type information. Finally, we confirmed the cell type by the selected gene marker list retrieved from previous literatures. The gene marker list used in this analysis is presented in Table S1 and the scRNA‐seq analysis pipeline using Seurat is released in github (https://github.com/zjh1224552972/PD_scRNAseq). Coefficient of variation of the counts per million (CPM) values used to identify the most variable gene sets across different cell clusters. The similarity between cells was estimated by PCA analysis using the highly variable genes. Cell clustering was visualized using Uniform Manifold Approximation and Projection (UMAP) for the top principal components.

### 2.3 Receptor‐ligand analysis

List of inferred receptor‐ligand pairs were obtained by searching for experimentally validated interactions (http://www.guidetopharmacology.org and http://dip.doe-mbi.ucla.edu/dip/DLRP.cgi). The receptors and ligands were filtered based on their CPM‐normalized expression. Only if the receptor and the ligand were both above 50 CPM in their respective cell types, the interactions were considered active.

**Highlights**

1. Landscape dynamics of cell populations during dialysis‐related peritoneal fibrosis.
2. Description of pathophysiology in peritoneal fibrosis at the single‐cell resolution level.
3. Elucidation of receptor‐ligand interaction networks between the fibroblasts and immune cells.

### 2.4 Statistical analysis

The differential expressed genes (DEGs) were identified by DESeq2 (v.1.10.1) and R (v.3.4.2). Genes were considered differentially expressed if they had an average |log2(fold change)| ≥ 0.58 and a Bonferroni‐adjusted *p* value < 0.05 (FDR < 0.05). Continuous variables were compared between two groups using the Mann–Whitney *U* test for nonnormally distributed variables and Student's *t‐*tests for normally distributed variables. Two‐tailed *p* values < 0.05 were considered significant.

### 2.5 Functional analysis of differentially expressed genes

The selected differentially expressed genes (DEGs) were submitted into the Gene Ontology (GO), KEGG, and Reactome enrichment analyses. GO and KEGG enrichment analyses were performed by using the online Database for Annotation, Visualization and Integrated Discovery (DAVID) tool (Version 6.8; http://david.abcc.ncifcrf.gov). The results of GO and KEGG analyses obtained from DAVID were visualized by GOplot R package and clusterProfiler R package, respectively. Enrichment information for Reactome pathway analysis was obtained from the ConsensusPathDB interaction database (http://ConsensusPathDB.org).

### 2.6 Pathway or Gene Set Enrichment Analysis

KEGG and GO signatures gene sets were downloaded from MSigDB database (https://www.gsea-msigdb.org/gsea/msigdb/collections.jsp#H). Gene Set Enrichment Analysis (GSEA) was performed using the GSEA software (v4.0.3) developed by Broad institute^16^ (https://www.gsea-msigdb.org/gsea/downloads.jsp) for annotation using pre‐ranking gene list, with MSigDB and permutation = 10,000 as parameters. The top‐ranking pathways or genesets with *p*‐value < 0.05 were used for downstream analyses.

## 3 RESULTS

### 3.1 Cell typing in normal peritoneum and PD effluent

**FIGURE 1:**
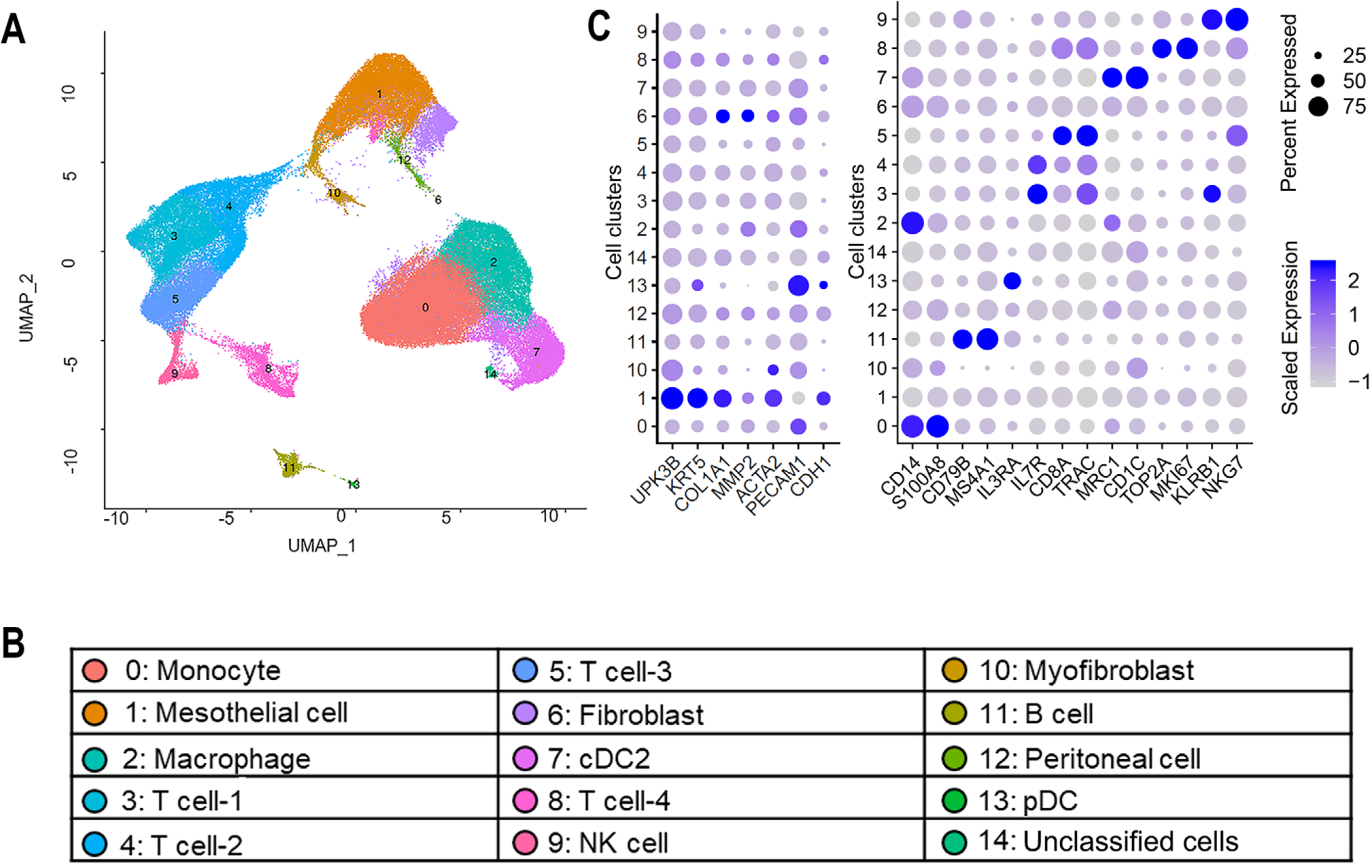
Diverse cell types delineated by single‐cell transcriptomic analysis. (A) The Uniform Manifold Approximation and Projection (UMAP) plot demonstrates main cell types in cells from all samples. (B) Cell types for UMAP plot are summarized in the panel below. (C) Dot map showing expression levels of specific markers in each cell type: cDC, conventional dendritic cell (previously called myeloid dendritic cell); pDC, plasmacytoid dendritic cell

To investigate the changes in cellular composition of human peritoneum in response to PD, we re‐analyzed the scRNA‐seq data. By using well‐known cell‐type markers (Table S1), we identified 15 main cell clusters including the mesothelial cell, fibroblast, myofibroblast, peritoneal cell, and kinds of immune cells such as monocyte, macrophage, B cell, T cell, dendritic cell (DC), and NK cell (Figure 1A–C). Here, we next focused on the heterogeneity of the same cell population in different subgroups in order to investigate the evolution of cell components of peritoneum in response to PD. Therefore, the cell types in normal peritoneum (healthy control, hc group), peritoneum from patients undergoing short‐term PD (st group), and peritoneum after long‐term PD (lt group) were profiled separately.

**FIGURE 2:**
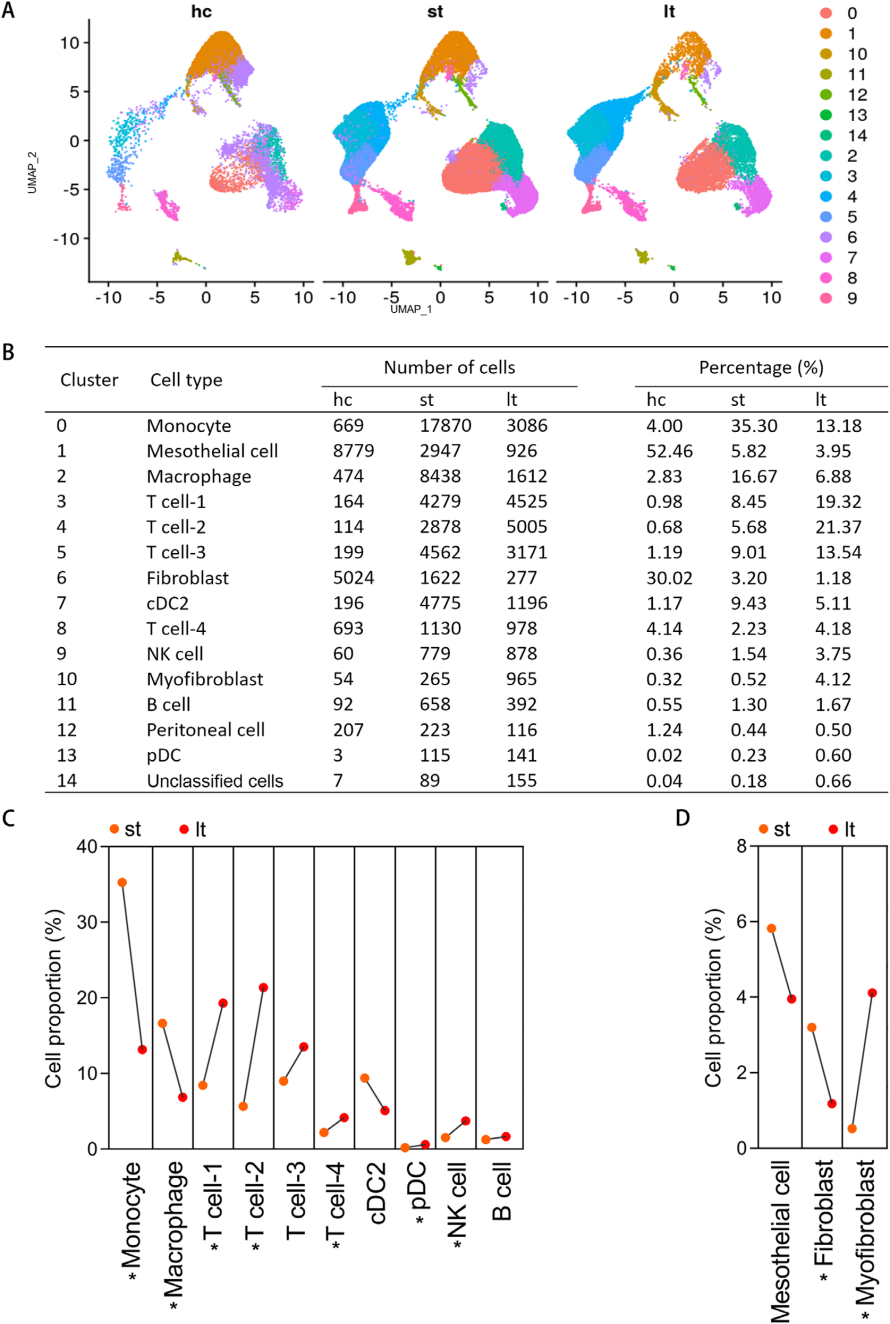
Single‐cell transcriptomic analysis delineates heterogeneous cell populations between normal human peritoneum, effluent of patients undergoing short‐term PD, and effluent of patients undergoing long‐term PD. (A) UMAP plot of healthy control group (hc), effluent of patients undergoing short‐term PD (st), and effluent of patients undergoing long‐term PD (lt) are displayed separately. (B) Percentages of assigned cell types in each group are summarized. (C) Line chart showing the changes in the proportions of cell populations in immune cells between st and lt groups. (D) Line chart showing the changes in the proportions of cell populations in mesenchymal cells between st and lt groups. ^*^ *p* < 0.05, Mann–Whitney *U* test

As visualized in UMAP plot (Figure 2A) and data graph (Figure 2B), cell components were quite different between st and lt groups. Compared with the st group, the proportions of monocytes, macrophage, and fibroblast in the effluent of the lt group were significantly decreased, while the proportions of T cell, pDC, NK cell, and myofibroblast were significantly increased (Figure 2C and D). The changes in the components of different cell types with the progress of PF suggested different roles of each cell played in different PF stage.

### 3.2 Single‐cell level transcriptomic changes of mesothelial cell and fibroblast in response to PD

**FIGURE 3:**
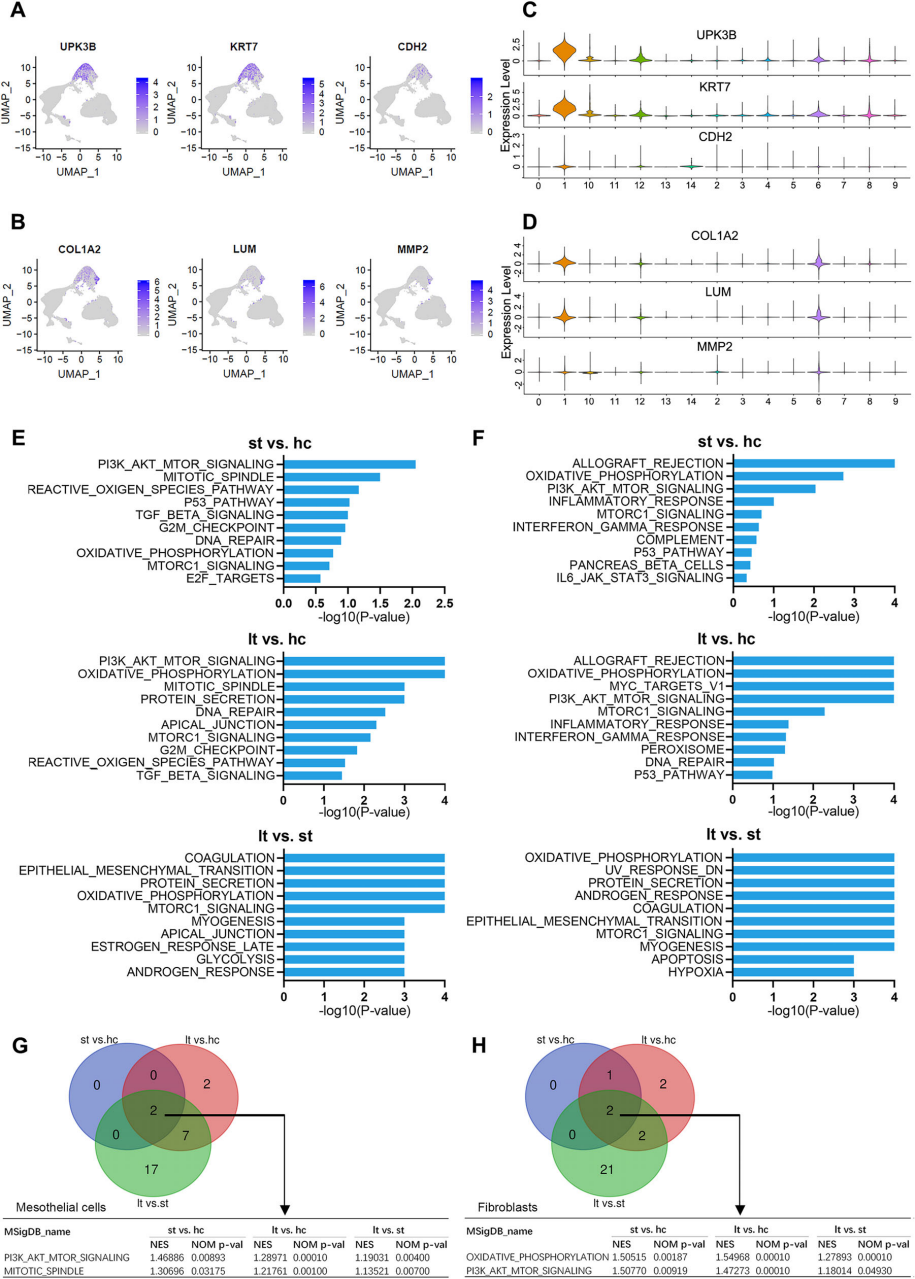
Differential expression analysis of the scRNA‐seq data identifies transition of characteristic genes in mesothelial cell and fibroblast as peritoneal fibrosis progresses. (A and B) Cell markers were used to label clusters by cell identity as represented in the UMAP plot. Mesothelial cell (A) and fibroblast (B) are shown. (C) Violin plots displaying the expression of well‐known specific markers for mesothelial cells across each group. (D) Violin plots displaying the expression of well‐known specific markers for fibroblasts across each group. (E) Gene set enrichment analysis (GSEA) was performed between hc, st, and lt group of mesothelial cells. Histograms show the top 10 gene sets enriched in the indicated group for each comparison. (F) GSEA was performed between hc, st, and lt group of fibroblasts. Histograms show the top 10 gene sets enriched in the indicated group for each comparison. (G) Venn diagram shows the intersection of significantly enriched gene sets from GSEA result of each comparison in mesothelial cells. Numbers in each area represent the number of significantly enriched gene sets. (H) Venn diagram shows the intersection of significantly enriched gene sets from GSEA result of each comparison in fibroblasts. Numbers in each area represent the number of significantly enriched gene sets

We compared gene expression between each group in two key parenchymal cell populations: mesothelial cell and fibroblast. The UMAP plots displayed the well‐recognized markers for mesothelial cells (Figure 3A) and fibroblasts (Figure 3B) for cells of all samples. Additionally, the expression levels of representative specific markers for mesothelial cells and fibroblasts in each group were shown in Figure 3C and D, respectively. The DEGs between hc, st, and lt groups in both mesothelial cells and fibroblasts were estimated and summarized in Tables S2 and S3, respectively. Gene Ontology enrichment analysis (GO analysis) of DEGs obtained from each comparison (st vs. hc and lt vs. hc) revealed enrichment of terms associated with extracellular matrix remodeling in both mesothelial cells (Figure S1, Table S4) and fibroblasts (Figure S2, Table S5) from patients with peritoneal dialysis, which was in consistent with the existing knowledge that the mesothelial cells and fibroblasts were involved in the progression of PF during dialysis. Further GSEA identified cell type–specific and disease progression‐specific hallmark gene sets relevant to PF in both mesothelial cells (Table S6) and fibroblasts (Table S7). The top 10 enriched gene sets in each comparison for mesothelial cells and fibroblasts were shown in the histograms of Figure 3E and F, respectively. Overall, the number of enriched gene sets progressively increased from st group to lt group in both mesothelial cells and fibroblasts, suggesting accumulation of aberrantly activated pathways and biological processes with increasing dialysis time. Comparing with the hc group, only the gene set of AKT/mTOR pathway was found to be enriched in mesothelial cells and fibroblasts from both st and lt groups (Figure 3G and H, Figures S3 and S4); meantime, the gene set of AKT/mTOR pathway was also enriched in both mesothelial cells and fibroblasts of lt group when comparing with that of st group, indicating there was progressively enhanced AKT/mTOR pathway activity in both mesothelial cells and fibroblasts along with the PF progresses. Additionally, in line with the existing theory, it is lt group rather than st group that showed significant enrichment of the epithelial‐mesenchymal transition (EMT) gene set (Figure 3E and F), suggesting conversion of cells from epithelial‐to‐mesenchymal phenotype is a gradual process during PF. In addition, we identified significantly enrichment of oxidative phosphorylation gene set in mesothelial cells and fibroblasts of st group in comparison with that of hc group; meanwhile, significantly enrichment of oxidative phosphorylation was also observed in both two cells of lt group when comparing with that of st group, implicating progressively altered glucose metabolism in both mesothelial cells and fibroblasts during PF progresses.

### 3.3 Elevated expression of immune genes in fibroblast in response to PD

**FIGURE 4:**
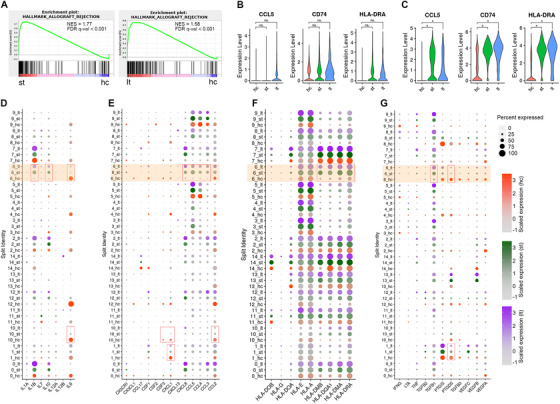
The scRNA‐seq analysis reveals distinct expression profiles of inflammatory genes in fibroblast and the other cell populations across each group. (A) Gene Set Enrichment Analysis was performed using the gene set of Allograft rejection for fibroblasts in both short‐term dialysis group and long‐term dialysis group compared with control group. (B and C) Violin plots of expression for select genes in mesothelial cells (B) and fibroblasts (C). (D–G) Expression of selected genes: (D) interleukins, (E) chemokines and receptors, (F) human leukocyte antigens, and (G) cytokines, separated by hc, st, or lt group. Dot size represents the percentage of cells expressing the corresponding gene, and dot color represents the average expression level. The orange‐colored blocks highlight the cluster 6 (fibroblasts). Each red box indicates there were significant differences (*p* < 0.05 and |log2(fold change) | ≥ 0.58, Student's *t*‐test) in the expression of corresponding genes in both comparisons (st vs. hc, lt vs. hc). For (B) and (C), the * symbol indicates *p* < 0.05 and |log2(fold change) | ≥ 0.58, Student's *t*‐test

Despite the shared mechanism mentioned above, perhaps surprisingly, GSEA identified that the allograft rejection was the most significantly enriched gene set in fibroblasts of both st and lt groups when comparing with fibroblasts of hc group (Figures 3F and 4A). Similarly, GO analysis revealed that DEGs of fibroblasts in st and lt groups were enriched in immune response processes such as leukocyte migration, neutrophil activation, neutrophil‐mediated immunity, cell chemotaxis, and positive regulation of response to external stimulus (Figure S2). Furthermore, KEGG analysis also demonstrated a larger number of significantly enriched immune‐relevant pathways in the fibroblasts of both st and lt groups (Table S8, Figures S5 and S6). Collectively, these findings suggested that the fibroblasts obtained from patients received PD have obtained some functional characteristics like chemotaxis and immune response. For example, CCL5, a well‐known chemokine responsible for infiltration of monocytes and T cells, was just slightly increased in the mesothelial cells of lt group (Figure 4B). In contrast, CCL5 was significantly increased in the fibroblasts of both st and lt groups (Figure 4C). Similarly, CD74 and HLA‐DRA, two important genes involved in antigen presentation and immune response, were also significantly increased in the fibroblasts from st and lt groups (Figure 4C), but not in mesothelial cells (Figure 4B).

To further investigate the characteristics of fibroblasts from short‐term PD patients, we evaluated the expression of some other immune‐related genes in each cell of each group. The expression of interleukins, chemokines and chemokine receptors, human leukocyte antigens (HLAs), and a variety of cytokines in each cluster was summarized in Figure 4D–G, respectively. In other two parenchymal cells, the mesothelial cell (cluster 1) and myofibroblast (cluster 10), the expression of most genes did not change significantly (Tables S2 and S9). In contrast, a number of genes, such as CCL3, CCL4, CCL5, CXCL8, IL‐1β, IL‐10, HLA‐DMA, HLA‐DMB, HLA‐DQA1, HLA‐DRA, and TGF‐β1 were upregulated; meanwhile, IL‐6 and PTGDS were decreased significantly in fibroblasts (cluster 6, highlighted in orange box) from PD patients (st group, lt group) related to those from healthy patients (hc group), indicating that fibroblasts with both immune recruitment function and pro‐inflammatory effect could occur in the very early stage of peritoneal dialysis. Additionally, there were no significant changes of VEGF in fibroblast during PD, suggesting fibroblast may not have strong effects in angiogenesis.

### 3.4 Profiling of cell receptor‐ligand interactions identified multiple cells targeted by fibrosis

**FIGURE 5:**
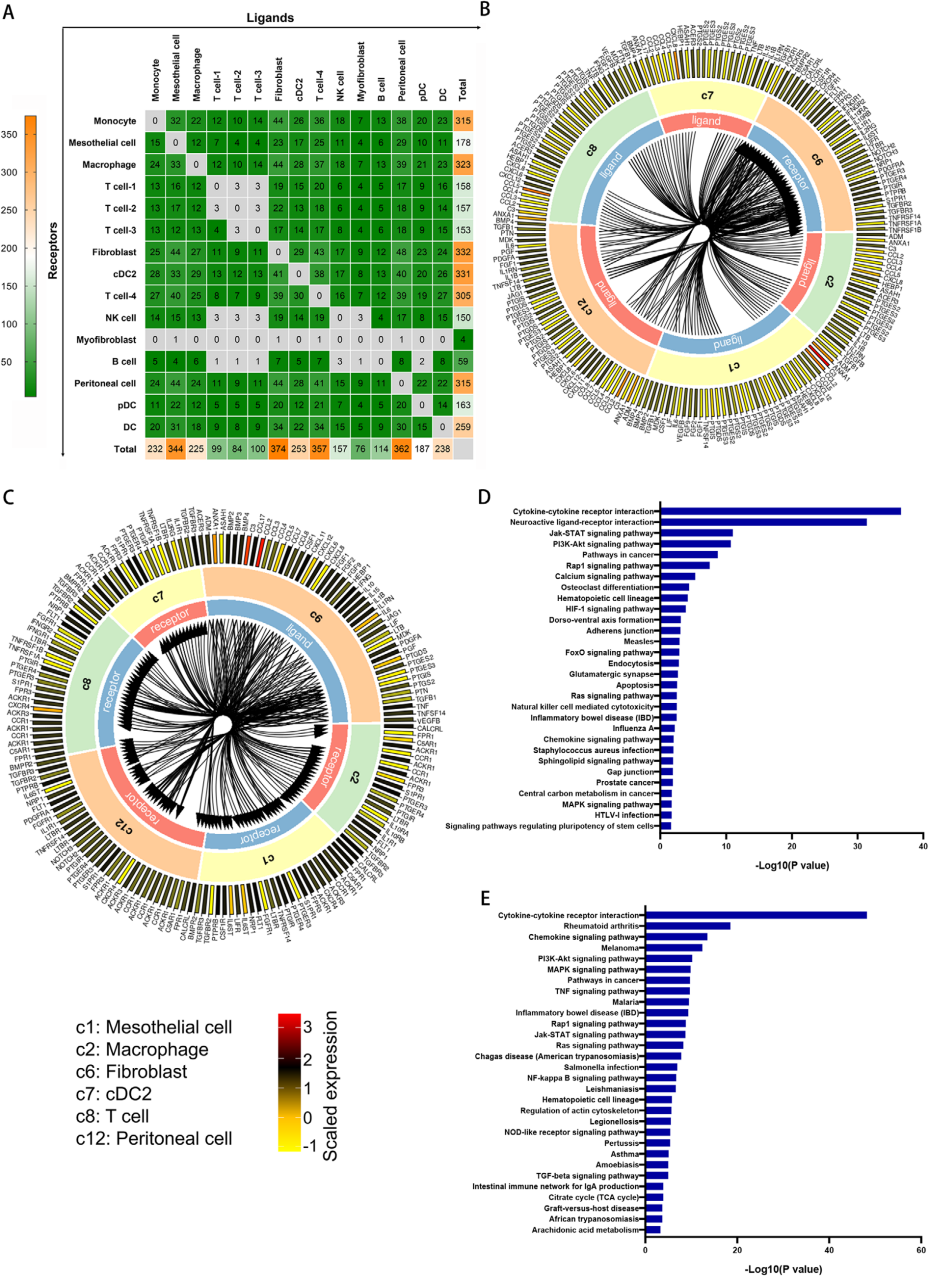
Putative receptor‐ligand interactions between fibroblast and multiple cell populations. (A) Heatmap showing the number of ligands and receptors estimated to have arisen at each paired cell populations. The *X* axis represents the numbers of ligands, and *Y* axis represents the numbers of receptors for the corresponding cell populations. (B) Circular plot displays the impact of five cell populations on fibroblast. Lines originate at the ligand and connect to its receptor as indicated by the arrowhead. (C) Circular plot displays the impact of fibroblast on five cell populations. Lines originate at the ligand and connect to its receptor as indicated by the arrowhead. c1 represents mesothelial cell, c2 represents macrophage, c6 represents fibroblast, c7 represents cDC, c8 represents T cell, and c12 represents peritoneal cell. The outer ring represents ligands and receptors, and colors correspond to the average expression level for the gene. (D) Functional enrichment analysis with GO biological processes was performed for downstream pathways of the receptors. (E) Functional enrichment analysis with GO biological processes was performed for downstream pathways of the ligands

Our knowledge of the intercellular communication between each cell type during PD is extremely limited. The scRNA‐seq provides a new opportunity to advance our understanding of the landscape of receptor‐ligand interactions by analyzing gene expression in individual cell population. Paired cognate receptors and ligands of each cell type present in short‐term PD cells were indicated in Figure 5A, and we can find crosstalks with other cells were mainly observed in mesothelial cell, fibroblast, peritoneal cell, and a subtype of T cells. Due to the obvious change in fibroblast component during PD‐related PF, as well as the findings that fibroblast undergoes evolution related to immunity, we examined the connection between fibroblast and other cells. Unlike most previous studies that have focused on how the fibroblast was activated or transformed, we paid a more attention to the effect of fibroblast on other cells in samples from patients undergoing short‐term PD. We also further focused on specific cell types linked normal peritoneum and severely fibrotic peritoneum after long‐term PD that helps to uncover the initiative contribution of fibroblast during the progression of PD‐related PF. As shown in Figure 5B and C, we found intensive engagement between fibroblast and five other cell types, including macrophage, T cells, mesothelial cell, cDC, and peritoneal cell. Fibroblast expressed kinds of chemokines, the receptors of which were expressed within the immune cell population,^17^ indicating a potential mechanism for attracting leukocytes and macrophages during PF progression. GO analysis of the differently expressed receptors (Figure 5D) and ligands (Figure 5E) identified downstream pathways involved in this cellular‐interaction network. The ligands expressed from fibroblasts are mostly involved in the activation of multiple classic pathways, including NOD‐like receptor signaling pathway, Toll‐like receptor signaling pathway, TNF signaling pathway, and NF‐kappa B signaling pathway. Meanwhile, two signaling pathways in fibroblasts are mainly activated by other cell populations, including the Jak‐STAT signaling pathway and the PI3K‐AKT signaling pathway.

## 4 DISCUSSION

Compared with bulk sequencing, scRNA‐seq is a promising technique that allows the analysis of transcriptome information at single‐cell resolution. By analyzing the scRNA‐seq data, we described the changes in cellular composition in early and late stages of PF. Functional annotation and enrichment analysis in fibroblast and mesothelial cell revealed critical biological mechanisms relevant to PF as well as their cell type–specific and disease process–specific characteristics. Our analysis suggests the presence of fibroblast with pro‐inflammatory phenotype is an extremely early event in patients undergoing peritoneal dialysis, which maintains at a stable degreed until the end stage of PF. The intensive signal interactions between fibroblast and other cells suggest an important role of fibroblast as in situ pro‐inflammatory cells in the initiation of fibrotic process and the transformation of parenchymal cells.^2^

Although the mechanism involved in PF remains not fully clear, it has been known that accumulation of activated fibroblasts is one of the key steps in fibrotic progression.^18^ Despite providing structural framework for tissues, the alterations in phenotype of fibroblasts may occur during different pathological conditions of different diseases.^2^, ^19^, ^20^ Besides, even the same tissue can be populated with several fibroblast subsets with distinct features.^21^ During PF, there are several sources of pathological fibroblasts, including mesothelial cells, resident fibroblasts, and even bone marrow–derived fibrocytes.^22^ According to current theories, during peritoneal dialysis, the abnormal activated fibroblasts, both in situ and recruited, eventually differentiate into “myofibroblast” that plays a vital role in secreting extracellular matrix.^23^, ^24^ However, despite as a source of myofibroblasts, their other roles in PF were still poorly understood. Although some studies have addressed that they participated in epithelial‐mesenchymal transition, angiogenesis and recruitment of immune cells, given the unique immune environment of abdominal cavity, improved understanding of peritoneal fibroblast is much needed.

The sources of inflammatory cytokines during the progression of peritoneal fibroblast have not been fully elucidated. Usually, the cytokines that control the recruitment of peripheral blood monocyte are released locally at sites of inflammation,^25^, ^26^ therefore they are previously believed to derive largely from the cytokine‐stimulated mesothelial cells.^27^, ^28^ In recent years, it gradually comes to light that fibroblasts may also serve as an important source of chemokines, but the role of fibroblasts as a chemokine source during PF has not yet been elucidated.^29^ According to the cellular‐level expression profiles of this study, although the expression of some interleukins and chemokines in mesothelial cells was evaluated in PD samples, their expression levels were relatively modest among all cell populations. Compared with mesothelial cells, the fibroblasts seem to be able to express more chemokines under the stimulation of peritoneal dialysis. In samples from PD patients, the expression levels of interleukins, cytokines, and chemokines in myofibroblast were much lower than those in both mesothelial cells and fibroblasts, which was consistent with its role as a terminal differentiated cell in PF. These findings have revealed the important role of fibroblasts as an initiator of inflammatory reaction and a source of recruiting immune cells.

Our results confirm some previously experimental results. CCL5 is a chemoattractant with broadly attractive effects for monocytes, T lymphocytes, NK cells, basophils, and eosinophils.^30^ Previously experimental study demonstrated that human peritoneal fibroblasts could synthesize CCL5 in response to inflammatory mediators presented in the inflamed peritoneal cavity.^31^ Our sing‐cell analysis revealed that CCL5 is one of the mostly upregulated chemokines in fibroblast in response to dialysis. Notably, CCL5 is mostly expressed in fibroblasts of short‐term dialysis patients, supporting our hypothesis that fibroblasts connect early PF to late PF through the function of recruiting inflammatory cells. Studies have also found that fibroblasts release chemokines CCL2 and IL‐8 (CXCL8).^32^ Our results showed a significant raising of IL‐8 level in fibroblasts from PD patients, and IL‐8 seems to be the most important interleukins released by fibroblasts. Compared with IL‐8, the expression level of CCL5 was relatively stable across healthy controls and PD groups. Besides the known factors, we revealed a landscape of secretory factors in fibroblasts, which demonstrated the ability of releasing much more factors compared with the current known. Interestingly, the expression of VEGF family showed a decreased tendency in the fibroblasts after PD stimulation; in contrast, the expression level of TGF‐β1 increased obviously, which was consistent with the hypothesis that the continuous activation of TGF‐β signaling pathway promotes the transformation of fibroblasts as appearing in the entire process of PF.

Dysregulated innate and adaptive immune responses are major contributors to PF.^7^ The whole process of inflammatory reaction is governed by a complex network of cytokines, chemokines, and molecules derived from pathogens and damaged cells. In the former part of this study, we constructed a receptor‐ligand based intercellular communications network at a single cellular resolution. We identified intensive interaction receptor‐ligand interactions among multiple cell populations, such as T cells, macrophages, conventional dendritic cells, mesothelial cells, and fibroblasts. The ligands expressed from fibroblasts are mostly involved in the activation of multiple classic pathways, including NOD‐like receptor signaling pathway, Toll‐like receptor signaling pathway, TNF signaling pathway, and NF‐kappa B signaling pathway. Meanwhile, two signaling pathways in fibroblasts are mainly activated by the other cell populations, including the Jak‐STAT and PI3K‐AKT signaling pathways. For the first time, our discoveries described the intercellular communications network that can potentially affect cellular behavior and progression of PF, enabling advances in cancer and inflammation biology, providing new insights into the mechanism of PF, from the view of intercellular synergistic interactions.

By using scRNA‐seq technology, Si et al.^15^ have revealed that hyperglycolytic metabolism in human mesothelial cells is responsible for peritoneal fibrogenesis, which provides a new therapeutic target to combat PF. Although our scRNA‐seq data were retrieved from theirs, our present study is essentially different. First, Si et al. focused on mesothelial cells in PD, while we focused on the dynamic changes of cell composition and cellular function along with the progress of PD, especially on that of fibroblasts. We provided the detailed description of pathophysiology in PF at single‐cell resolution level. Second, despite there were multiple cell populations in the samples, Si et al. just used the transcriptome data of the mesothelial cells. They used scRNA‐seq technology as a way of sorting mesothelial cells, without further analyzing other cells, such as immune cells and fibroblasts. It is mainly because they focused on the mechanism involved in promoting mesothelial‐to‐mesenchymal transition of the mesothelial cells. In comparison, our study further made a much fuller use of the scRNA‐seq data. Based on the single‐cell transcriptome data of multiple cell types, we described the receptor‐ligand interactions among multiple cell populations and revealed the intercellular interactions during PD for the first time.

In conclusion, our analysis based on scRNA‐seq data revealed dynamic changes in the cellular composition and function of individual cell populations during PD. We demonstrated that the phenotype of fibroblasts has changed significantly just in response to short‐term dialysis. The characteristics of gene expression in fibroblasts from PD patients support a central proinflammatory role of fibroblasts. This present analysis offers a promise for the generation or exclusion of hypotheses regarding the function of fibroblasts that emerge during diseases. Moreover, these molecular signatures also begin to reveal processes that may underlie the crosstalk between fibroblasts and other multiple cell populations during the process PF.

## ETHICS APPROVAL AND CONSENT TO PARTICIPATE

The study has been approved by the research ethics committee of the Third Affiliated Hospital of Sun Yat‐sen University (IRB Number: [2020]02‐166).

## CONSENT FOR PUBLICATION

Contents for publication were obtained from all patients.

## AUTHOR CONTRIBUTIONS

Ning Na, Tong Zheng, and Jinhua Zhang designed experiments, analyzed data, and interpreted results. Jinhua Zhang, Yuxian Chen, and Tufeng Chen analyzed all the data and wrote this manuscript. Bin Miao, Zuofu Tang, Xiao Hu, and You Luo performed data acquisition. Tong Zheng reviewed and edited the manuscript. Ning Na coordinated and directed the project. All authors read and approved the final manuscript.

## CONFLICT OF INTEREST

The authors declare that they have no conflict of interest.

## Supporting information



Supporting Information

Supporting Information

Supporting Information

Supporting Information

Supporting Information

Supporting Information

Supporting Information

Supporting Information

Supporting Information

Supporting Information

## Data Availability

The data that support the findings of this study are available in the Supporting Information Materials of this article.
