# Supplementary material for: Single‐cell transcriptomics provides new insights into the role of fibroblasts during peritoneal fibrosis
Source: Clin Transl Med. 2021 Feb 26;11(3):e321. doi: 10.1002/ctm2.321 (PMC7908046; doi:10.1002/ctm2.321)
Supplement: Supplementary file 1 — Supporting Information [file CTM2-11-e321-s001.pdf]

Supplemental Figure 1

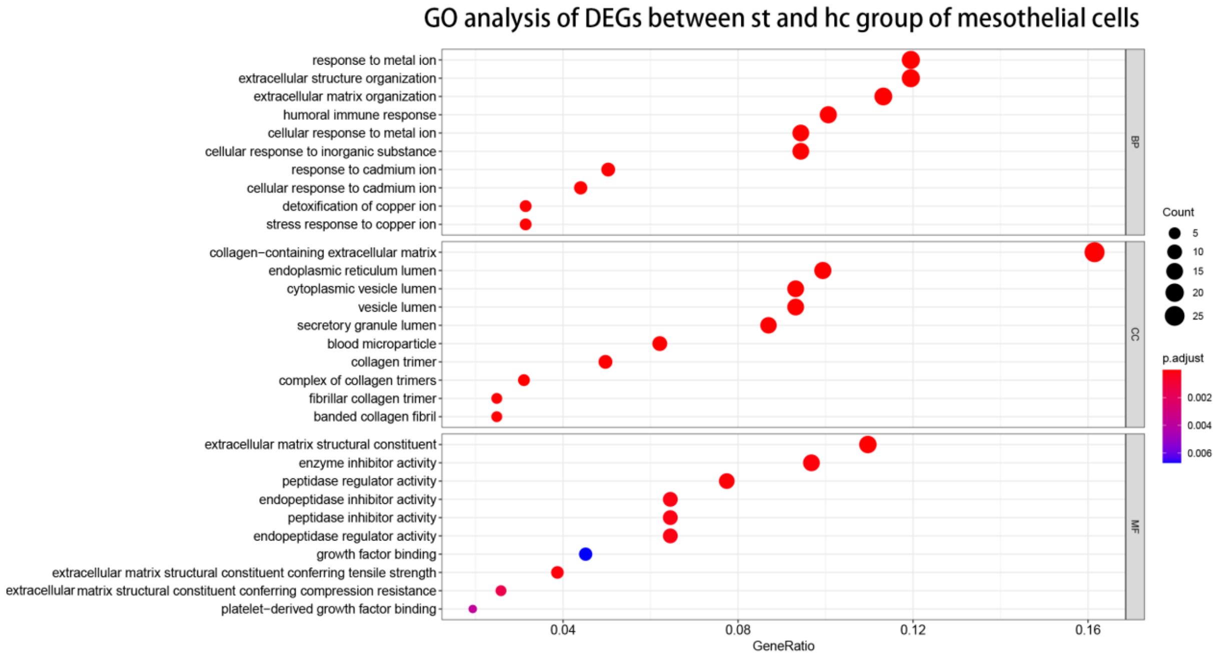

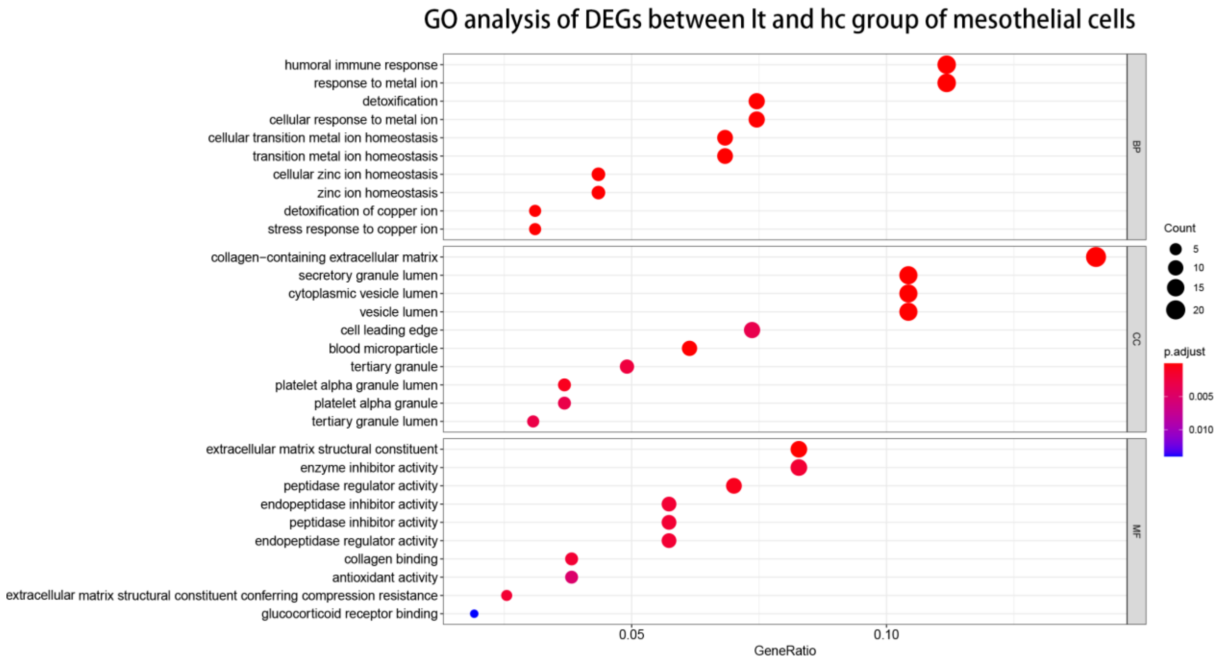

Supplemental Figure 1. Gene Ontology enrichment analysis (GO analysis) of DEGs (st vs. hc and lt vs. hc) in mesothelial cells.

Supplemental Figure 2

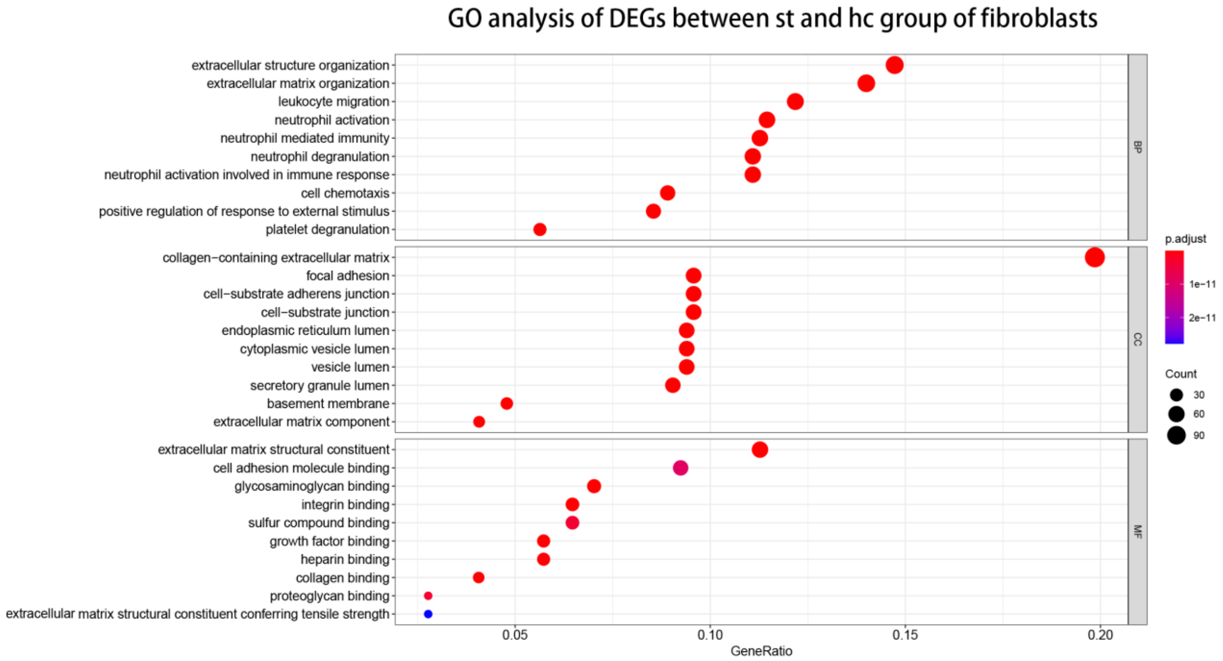

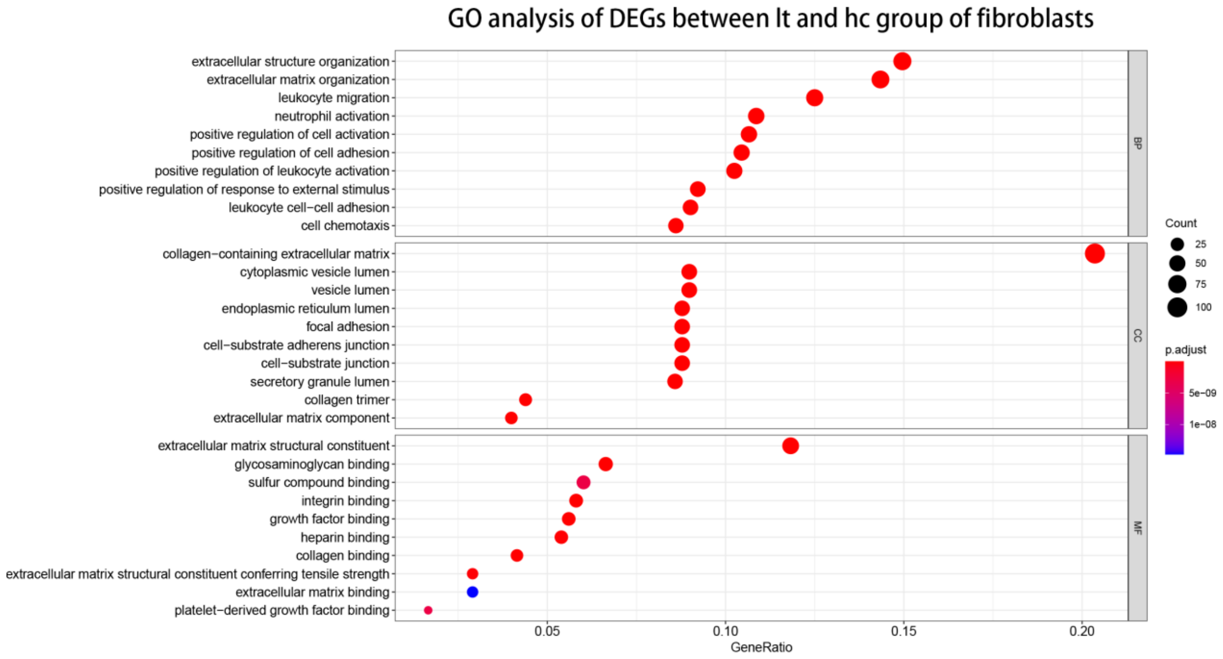

Supplemental Figure 2. Gene Ontology enrichment analysis (GO analysis) of DEGs (st vs. hc and lt vs. hc) in fibroblasts.

Supplemental Figure 3

Enriched Gene Sets of Mesothelial cells

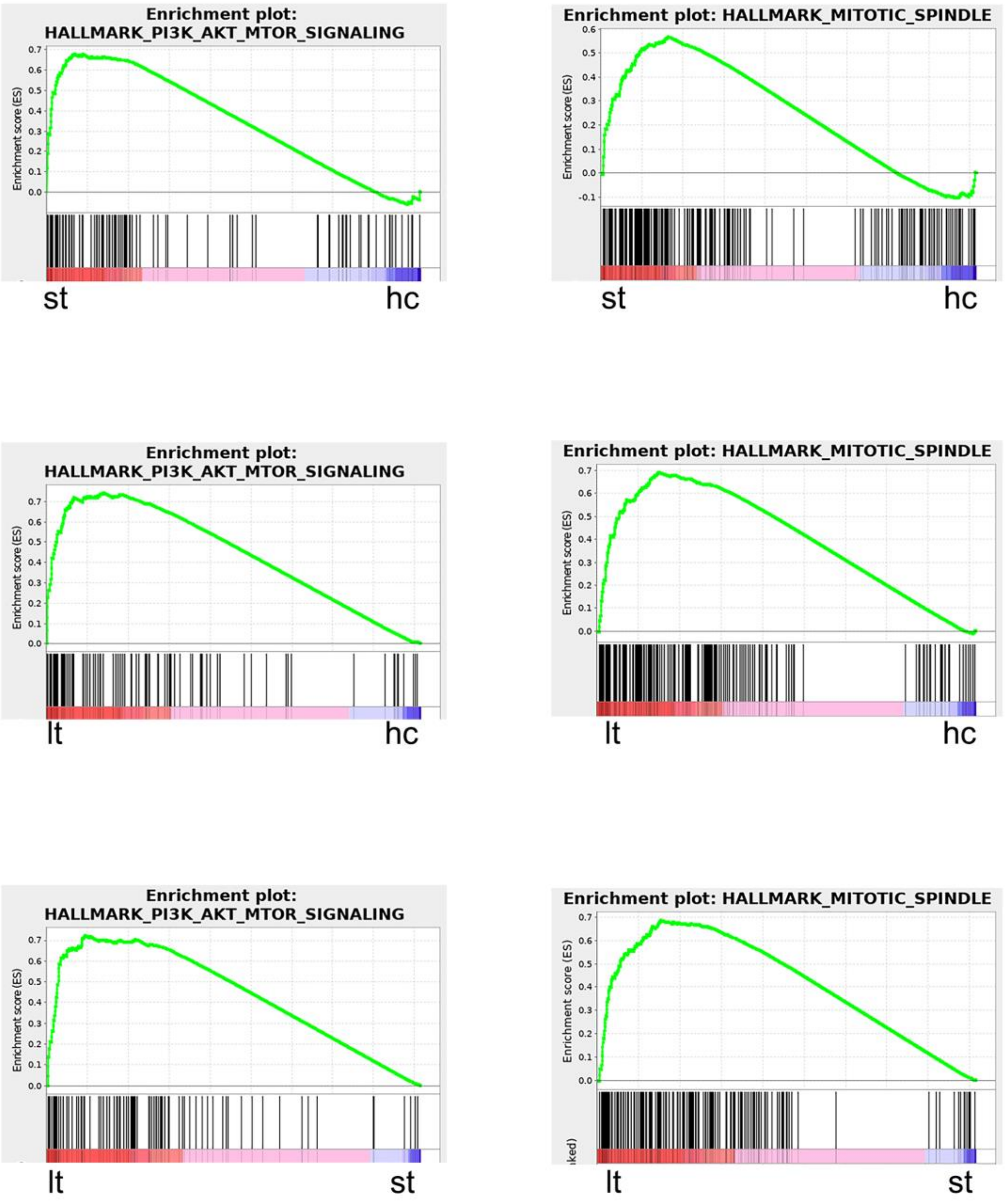

Supplemental Figure 3. Intersection gene sets of significantly enriched hallmark sets from GSEA result of each comparison in mesothelial cells corresponding to Figure 3G.

Supplementary Figure S4

Enriched Gene Sets of Fibroblasts

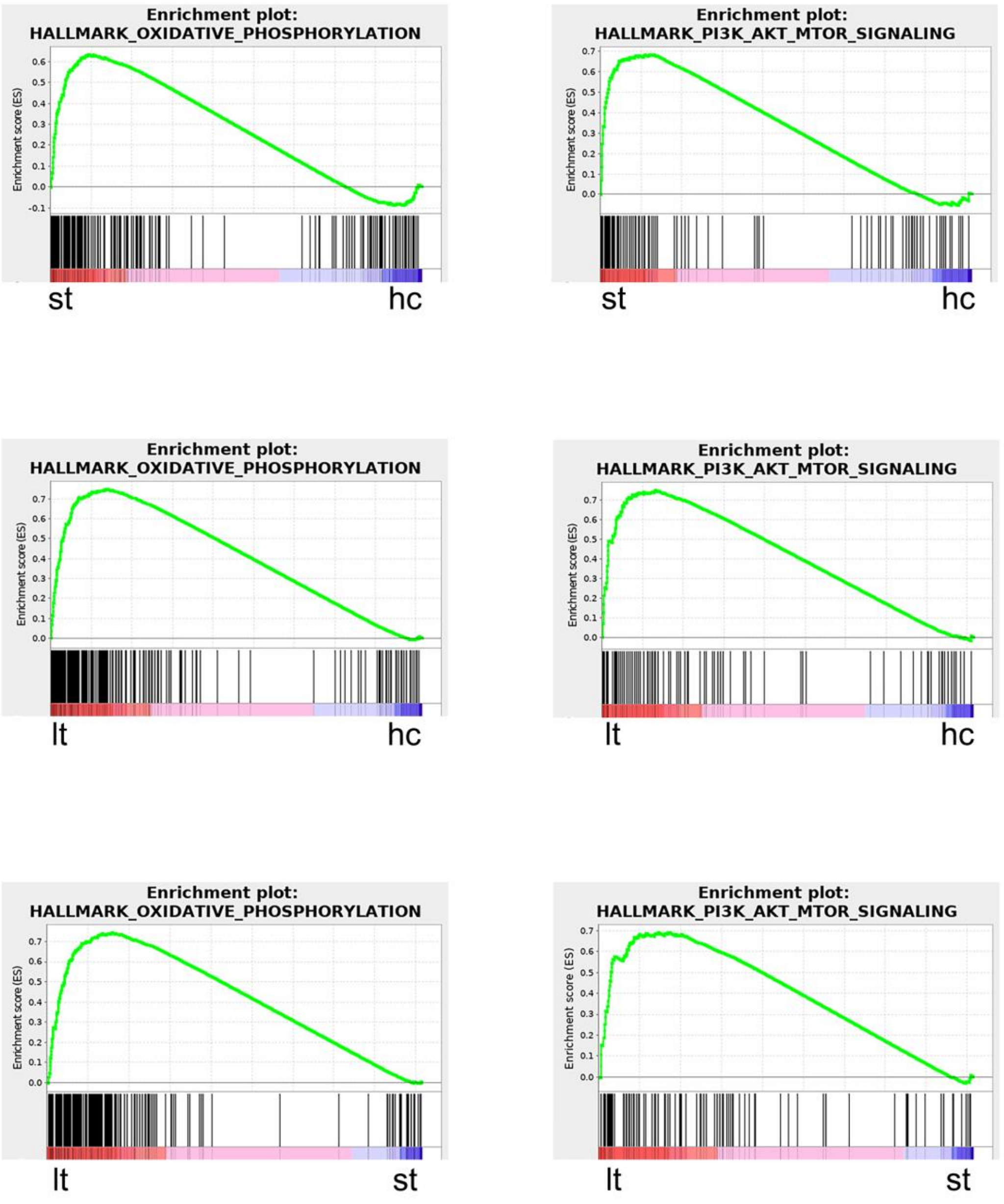

Supplemental Figure 4. Intersection gene sets of significantly enriched hallmark sets from GSEA result of each comparison in fibroblasts corresponding to Figure 3H.

Supplemental Figure 5

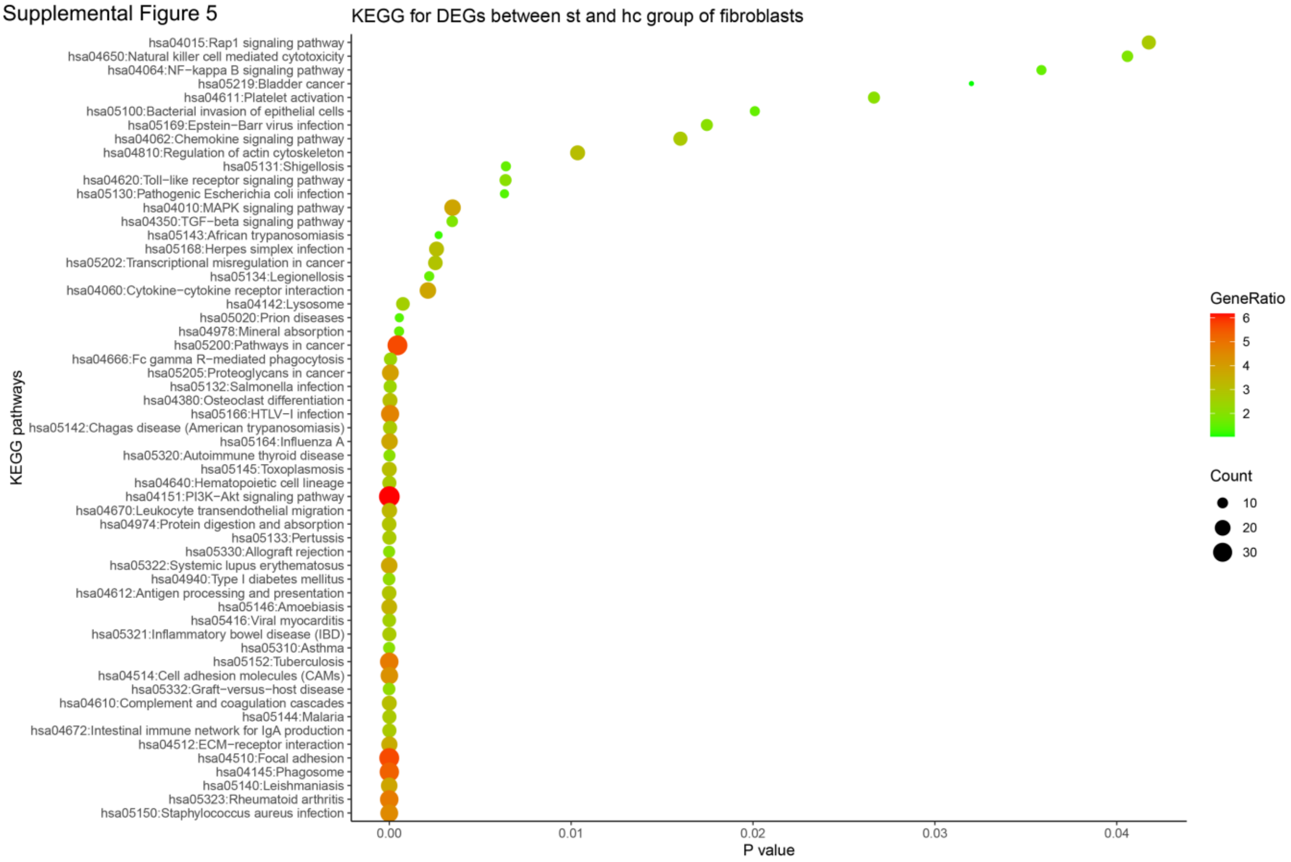

Supplemental Figure 5. Enriched pathways in KEGG analysis of DEGs from comparison between st and hc group in fibroblasts.

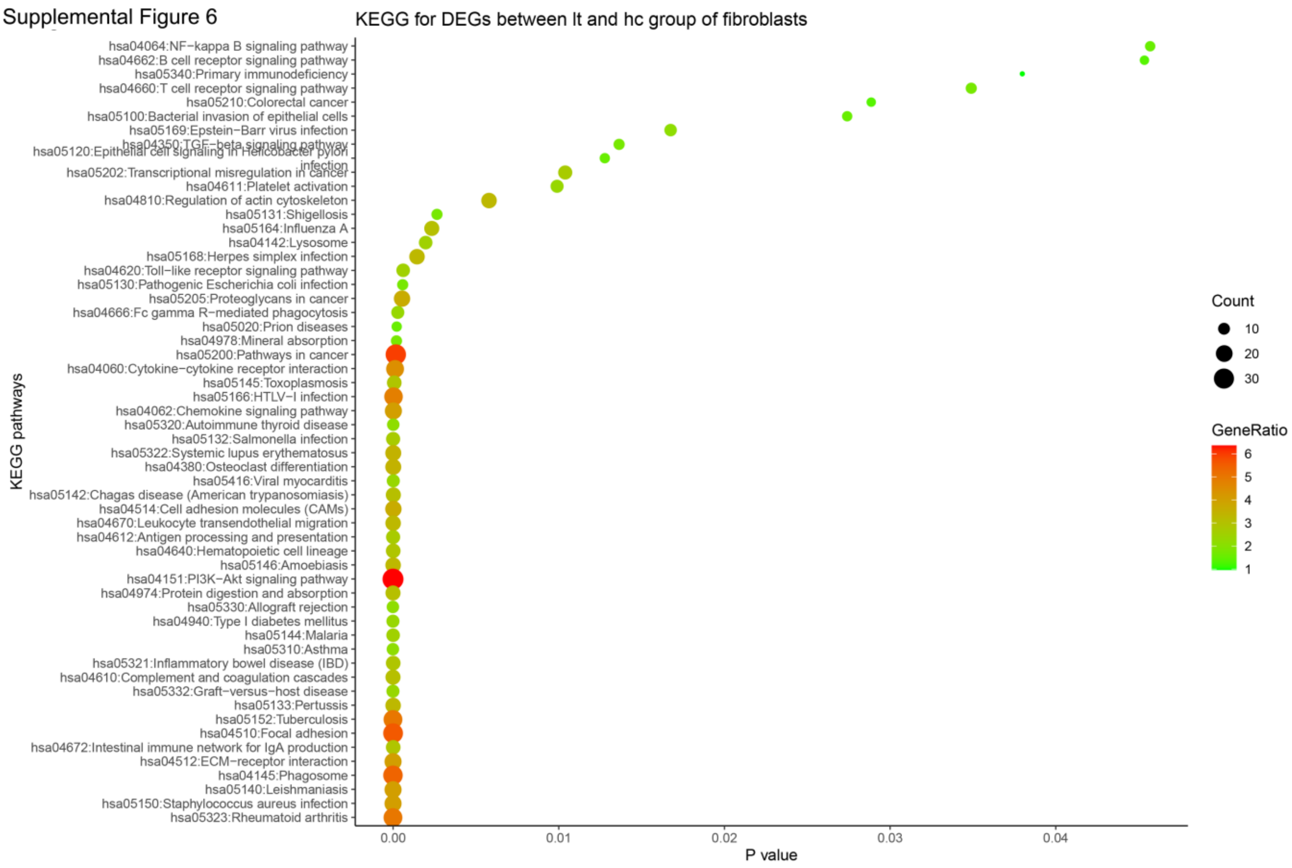

Supplemental Figure 6. Enriched pathways in KEGG analysis of DEGs from comparison between lt and hc group in fibroblasts.
